# Editorial: Targeting the tumor microenvironment for effective treatment of gastrointestinal cancers

**DOI:** 10.3389/fendo.2025.1600639

**Published:** 2025-04-30

**Authors:** Michela Roberto, Giulia Arrivi, Donatella Delle Cave

**Affiliations:** ^1^ Oncology Unit, Policlinico Umberto I, Haematological, Oncological and Dermatological Department, Sapienza, University of Rome, Roma, Italy; ^2^ Oncology Unit, Department of Clinical and Molecular Medicine Sapienza University of Rome, Sant’Andrea University Hospital, Rome, Italy; ^3^ Institute of Genetics and Biophysics ‘Adriano Buzzati-Traverso’ (IGB), National Research Council of Italy (CNR), Naples, Italy

**Keywords:** gastrointestinal cancer, tumor microenvironment, genetic alterations, target therapy, liquid biopsy

Gastrointestinal (GI) cancers, including colorectal, pancreatic, gastric, and liver cancers, are among the most common and deadly cancers worldwide. Despite advancements in screening and early detection, many patients are diagnosed at advanced stages, leading to high mortality rates and limited treatment options (1). GI cancers are genetically diverse, with specific mutations associated with different types. For example, colorectal cancers (CRCs) often harbor mutations in APC, KRAS, and TP53, while pancreatic cancers frequently show mutations in KRAS, CDKN2A, and SMAD4 (2, 3). Gastric cancers may exhibit amplifications of HER2 and mutations in CDH1, while hepatocellular carcinomas (HCC) often have mutations in CTNNB1 (β-catenin) or TP53, often in the context of chronic liver disease (4, 5). Recent research highlights the critical role of the tumor microenvironment (TME) in cancer progression and therapeutic resistance (6, 7). Tumor cells shape the TME by secreting growth factors and cytokines, which recruit and activate stromal components, remodel the ECM, and manipulate immune cells to support tumor growth and metastasis. Conversely, the TME influences tumor evolution by providing signals that enhance tumor cell plasticity, invasion, and resistance to therapies. For example, hypoxia in poorly vascularized tumor regions can select for more aggressive cell clones and upregulate survival pathways. These interactions demonstrate why treatments targeting tumor cells alone often fail (8, 9). In CRC, the TME significantly contributes to disease development and progression. A study by Xu et al. examined postoperative pathological tissues, clinical data, and pre-surgical tumor markers (such as CEA and CA199) from 268 CRC patients (stages I-IV). The study assessed the expression of inducible T-cell co-stimulator (ICOS), CD163 (a marker for M2 macrophages), and Foxp3 (a marker for regulatory T cells, Tregs) in cancerous, adjacent non-tumorous, and normal tissues. The study found that the expression levels of M2 macrophages, Tregs, and ICOS were higher in CRC tissues compared to adjacent and healthy tissues. As the tumor progressed, M2 macrophage and Treg levels increased, while ICOS expression decreased. Clinically, higher ICOS expression correlated with better long-term survival, and lower ICOS expression was associated with lymph-node and distant metastasis. These findings highlight the importance of the TME in CRC progression. Immune checkpoint inhibitors have expanded therapeutic options for CRC, improving patient outcomes, but microsatellite stable (MSS) CRC still shows poor response rates compared to microsatellite instable (MSI-H) CRC. To predict outcomes and response to immunotherapy, a study created an immune-related gene prognostic index (IRGPI) model based on immune-related gene patterns in normal and CRC tissues (Jin et al.). Survival-related genes such as FABP2, F2RL1, NR3C2, NR5A2, PPARGC1A, LGALS4, and XDH were identified, with NR5A2, PPARGC1A, and LGALS4 as independent prognostic genes. The IRGPI model showed strong predictive power for long-term survival, outperforming other models like tumor inflammatory signature (TIS) and tumor immune dysfunction and exclusion (TIDE). The model’s correlation with immune features indicated that high-risk CRC patients had decreased naïve B cells, plasma cells, and CD4+ memory T cells, along with increased M0 and M1 macrophages, with worse prognosis in patients with increased M1 macrophage infiltration. The liquid biopsy affords the valuable opportunity to stratify the patient’s risk and represents the most recent developments in the areas of precision and individualization of CRC treatment. A recent review provided a comprehensive analysis of advantages and disadvantages of all the liquid biopsy analytes, circulating tumor DNA (ctDNA) fragments, circulating tumor cells (CTCs), circulating tumor microRNAs (ctmiRNAs), circulating free DNA (cfDNA), exosomes, and metabolites, focusing on their clinical applicability in CRC management (He et al.). In addition to being a very useful real-time biopsy tool and a window into the molecular mechanisms underlying tumor cell pathogenicity and drug resistance, CTCs—tumor cells that separate from tumor tissue and enter the circulatory system—have the disadvantage of being challenging to detect in resected colorectal cancer. cfDNA refers to degraded DNA fragments released into the plasma, from necrotic and apoptotic tumor cells, circulating tumor cells, and exosomes. Detecting changes in the number of types of cfDNA in specific sequences can be used to identify not only common genes’ mutation, as KRAS and BRAF but also abnormal methylation patterns due to genetic and epigenetic changes that influence the development of CRC. Instead, the expression of miRNAs - a group of non-coding RNAs, 18–25 nucleotides in length - is related to tumor tissue type, degree of differentiation, aggressiveness, treatment prognosis, and other clinical biological characteristics. Exosomes reflect the metastasis development process and drug resistance, in fact exosomes guide the direction of tumor cell metastasis. On the one hand, tumor cells use exosomes as carriers to help them escape immune system surveillance and create a microenvironment suitable for tumor growth. The main disadvantage of using exosomes is the lack of a consensus on the technology to be used to detect them. Among upper GI, gallbladder cancer and gastric cancer (GC) are very aggressive, poor prognosis cancers which are often diagnosed at advanced stages. A study by Yuan et al. investigated the role of ASPH expression in bile duct cancer, finding that ASPH promotes gallbladder cancer development. Higher ASPH expression correlated with poorer histological differentiation, larger tumor size, advanced TNM stage, lymph node metastasis, and reduced survival. ASPH also contributed to immune evasion by affecting T cell and B cell function and weakening key immune pathways. Moreover, tumors with high ASPH expression showed resistance to gemcitabine and paclitaxel, suggesting that targeting ASPH could provide a therapeutic opportunity. GC, the fifth most common cancer worldwide and the fourth leading cause of cancer-related deaths, also requires better diagnostic biomarkers. The ability of GC to escape regulatory cell death mechanisms contributes to its aggressive nature. Sun et al. developed a new model called the regulatory cell death index (RCDI) signature, which includes cell-cycle regulatory genes like NOTCH3, BRCA1/2, and TGFB2. Using RNA-seq data from 1292 GC patients, the study found that high RCDI scores were associated with immune cell abundance and worse immunotherapy outcomes. The RCDI signature could serve as a valuable biomarker for predicting treatment efficacy and patient prognosis in GC (Sun et al.). In conclusion, integrating molecular profiling with immune and microenvironmental insights is crucial for improving prognosis and expanding treatment options for GI cancer patients. Ongoing research should focus on refining biomarkers, validating their clinical utility, and developing targeted therapies that overcome resistance mechanisms and enhance patient outcomes.

## References

[1] Arnold M Abnet CC Neale RE Vignat J Giovannucci EL McGlynn KA. Global burden of 5 major types of gastrointestinal cancer. Gastroenterology. (2020) 159:335–49.e15. doi: 10.1053/j.gastro.2020.02.068 32247694 PMC8630546

[2] Adamopoulos C Delle Cave D Papavassiliou AG. Inhibition of the RAF/MEK/ERK signaling cascade in pancreatic cancer: recent advances and future perspectives. IJMS. (2024) 25:1631. doi: 10.3390/ijms25031631 38338909 PMC10855714

[3] Delle Cave D Mangini M Tramontano C De Stefano L Corona M Rea I. Hybrid biosilica nanoparticles for *in-vivo* targeted inhibition of colorectal cancer growth and label-free imaging. IJN. (2024) 19:12079–98. doi: 10.2147/IJN.S480168 PMC11585298 39583322

[4] Wang K. Whole-genome sequencing and comprehensive molecular profiling identify new driver mutations in gastric cancer. Nat Genet. (2014) 46:573–82. doi: 10.1038/ng.2983 24816253

[5] Llovet JM Kelley RK Villanueva A Singal AG Pikarsky E Roayaie S. Hepatocellular carcinoma. Nat Rev Dis Primers. (2021) 7:6. doi: 10.1038/s41572-020-00240-3 33479224 PMC13537433

[6] Yokobori T Nishiyama M. TGF-β Signaling in gastrointestinal cancers: progress in basic and clinical research. JCM. (2017) 6:11. doi: 10.3390/jcm6010011 28106769 PMC5294964

[7] Roberto M Arrivi G Di Civita MA Barchiesi G Pilozzi E Marchetti P. The role of CXCL12 axis in pancreatic cancer: New biomarkers and potential targets. Front Oncol. (2023) 13:1154581. doi: 10.3389/fonc.2023.1154581 37035150 PMC10076769

[8] Boyd LNC Andini KD Peters GJ Kazemier G Giovannetti E. Heterogeneity and plasticity of cancer-associated fibroblasts in the pancreatic tumor microenvironment. Semin Cancer Biol. (2022) 82:184–96. doi: 10.1016/j.semcancer.2021.03.006 33737108

[9] Quante M Varga J Wang TC Greten FR. The gastrointestinal tumor microenvironment. Gastroenterology. (2013) 145:63–78. doi: 10.1053/j.gastro.2013.03.052 23583733 PMC4012393

